# Exercise in advanced prostate cancer elevates myokine levels and suppresses in-vitro cell growth

**DOI:** 10.1038/s41391-022-00504-x

**Published:** 2022-02-12

**Authors:** Jin-Soo Kim, Dennis R. Taaffe, Daniel A. Galvão, Nicolas H. Hart, Elin Gray, Charles J. Ryan, Stacey A. Kenfield, Fred Saad, Robert U. Newton

**Affiliations:** 1 Exercise Medicine Research Institute, Edith Cowan University, Joondalup, WA 6027 Australia; 2 School of Medical and Health Sciences, Edith Cowan University, Joondalup, WA 6027 Australia; 3 Caring Futures Institute, College of Nursing and Health Sciences, Adelaide, SA 5042 Australia; 4 Centre of Precision Health, Edith Cowan University, Joondalup, WA 6027 Australia; 5 Medical School, University of Minnesota, Minneapolis, MN 55455 USA; 6 Departments of Urology and Epidemiology & Biostatistics, University of California San Francisco, San Francisco, CA 94143 USA; 7 Department of Urology, Centre Hospitalier de l’Université de Montréal, Montréal, QC H2X 3E4 Canada

**Keywords:** Translational research, Prostate cancer

## Abstract

**Background:**

Altering the systemic milieu through exercise has been proposed as a potential mechanism underlying exercise-driven tumour suppression. It is not yet known whether men with advanced prostate cancer can elicit such adaptations following a program of exercise. The purpose is to examine myokine levels of serum acquired from metastatic castrate-resistant prostate cancer (mCRPC) patients recruited to the INTERVAL-GAP4 trial before and after 6 months of exercise and its tumour-suppressive effect.

**Methods:**

Twenty-five men with mCRPC (age = 74.7 ± 7.1 yrs) were randomised to supervised multimodal (aerobic and resistance) exercise (EX) or self-directed exercise control group (CON). Body composition was assessed using dual-energy x-ray absorptiometry (DXA), and fasting blood in a rested state was collected at baseline and at 6 months. Serum levels of myokines (SPARC, OSM, decorin, IGF-1, and IGFBP-3) were measured. Serum was applied to the prostate cancer cell line DU145, and growth was assessed for 72 h.

**Results:**

No significant change in body composition was observed. Adjusted serum OSM (*P* = 0.050) and relative OSM (*P* = 0.083), serum SPARC (*P* = 0.022) and relative SPARC (*P* = 0.025) increased in EX compared to CON. The area under curve (AUC) over 72 h showed a significant reduction in DU145 growth after applying post-intervention serum from the EX vs CON (*P* = 0.029).

**Conclusion:**

Elevated myokine expressions and greater tumour-suppressive effects of serum after 6 months of periodised and autoregulated supervised exercise was observed in men with mCRPC. Exercise-induced systemic changes may slow disease progression in men with advanced prostate cancer.

## Introduction

Exercise has been established as effective in improving physical function and supportive care outcomes for cancer patients, including those with advanced disease [1]. Furthermore, epidemiological studies of prostate cancer patients consistently report a positive association between increased physical activity levels and reduced risk of prostate cancer-specific mortality [2] and disease progression [3]. However, the causality of exercise-induced reduction of patient mortality and mechanisms of tumour suppression has not been thoroughly investigated in men with advanced prostate cancer [4, 5]. Accordingly, a global multi-centred Phase III randomised controlled exercise trial, INTERVAL-GAP4 [6], recruiting men with advanced prostate cancer is currently ongoing to examine the effect of exercise medicine on clinical outcomes, principally overall survival and disease progression, and the potential mechanisms by which exercise influences tumour biology.

Exercise induces multiple physiological changes, including alteration in cell-free and soluble molecules in the circulatory system known to have tumour-suppressive effects [5]. This has been further demonstrated in studies in which resting serum acquired after long-term exercise programs or exercise-conditioned serum obtained after a single bout of exercise applied to cancer cell lines produces substantial suppression of growth [7–13], with evidence supporting the involvement of exercise-induced serological insulin-like growth factor-1 (IGF-1) axis alteration in prostate cancer cell growth suppression [7, 11–13]. In our recent report [14], we showed suppression of androgen-independent prostate cancer cell line DU145 growth by applying serum acquired from patients with localised prostate cancer undergoing androgen deprivation therapy (ADT) following a 3-month exercise intervention and observed alterations in circulating cell-free/soluble factors compared to a pre-trained state, suggesting a potential of exercise in prostate cancer suppression [5].

Skeletal muscle has been identified as an endocrine organ and elicits health-related benefits by producing cytokines termed myokines, especially during exercise [15]. Furthermore, in vivo and in vitro application of myokines, such as irisin [16–19], decorin [20–24], interleukin-6 (IL-6) [25, 26], interleukin-15 (IL-15) [27, 28], secreted protein acidic and rich in cysteine (SPARC) [29–32], and oncostatin M (OSM) [33–35], have reduced the growth and migration of various types of cancer cell lines, including prostate [19, 20, 24–27, 30]. In addition, reduced myostatin expression counters the development of cachexia and may also contribute to tumour suppression by increasing irisin production [5]. However, despite the preclinical evidence, myokines are only considered as a *potential* molecular player for exercise-induced cancer suppression [5].

Although myokine expression in the non-cancer population is well documented [15], there has been no investigation of myokine expression and tumour-suppressive effects of exercise-conditioned serum in advanced prostate cancer patients who have undergone, and continue to receive, a range of cancer therapies inclusive of androgen blockade. Given the high disease load of these patients, with metastases and a shortened survival time, in addition to their castrate-resistant status underpinning an altered endocrine environment, it is important to determine if they can respond to exercise with the development of a more anti-tumour systemic milieu. Thus, in this study, we investigated resting serum myokine levels (irisin, decorin, IL-6, IL-15, SPARC, OSM, and myostatin) and growth hormone levels (IGF-1 and IGFBP-3) over the initial 6-month period of 2-year exercise intervention vs control group in metastatic castrate-resistant prostate cancer (mCRPC) patients. We also evaluated the potential exercise-induced tumour-suppressive effect by comparing serum acquired at baseline and after 6 months to the prostate cancer cell line DU145. We hypothesised that despite the heavy disease load and continuous treatment in this patient group (mCRPC), 6 months of supervised aerobic and resistance exercise would alter circulatory myokine levels and that exercise-conditioned serum obtained from the patients would reduce the growth of the prostate cancer cell line DU145.

## Materials/subjects and methods

### Participants and exercise program

**Fig. 1:**
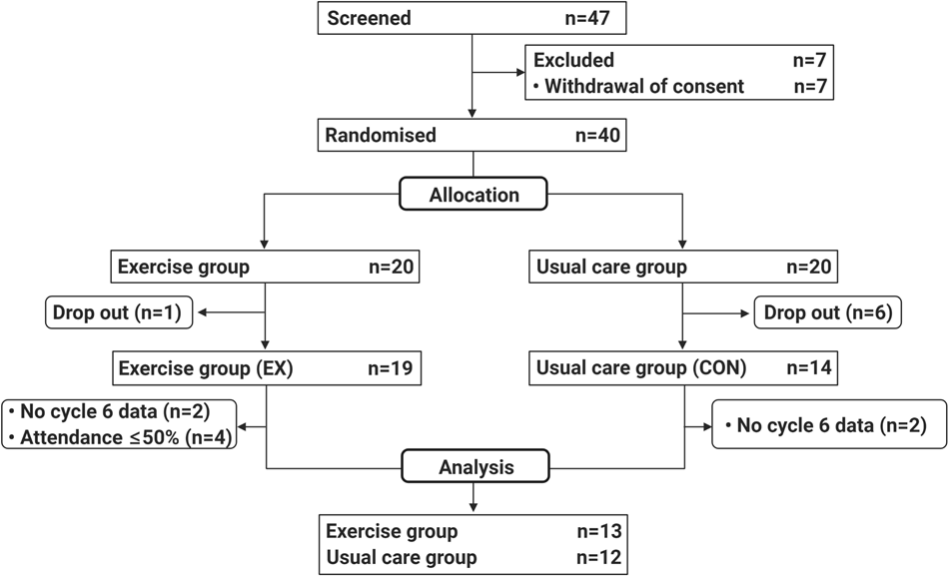
Consort diagram. Cycle 6 indicates end of the initial 6-month phase of the INTERVAL GAP4 trial. Due to COVID-19 restrictions, patients who could not visit the centre for cycle 6 assessments were excluded from the analysis.

Serum was collected from 25 men with mCRPC (EX, *n* = 13; CON, *n* = 12) who were recruited for the INTERVAL-GAP4 trial (Clinical Trials Registry: NCT02730338) [6] from March 2016 to May 2020 at the Exercise Medicine Research Institute (EMRI; Edith Cowan University (ECU); WA, Australia) which was used for analysis (Fig. 1). The recruitment and randomisation of patients were undertaken as previously described [6]. Briefly, patients who had been identified as mCRPC (adenocarcinoma of the prostate with systemic metastatic disease despite castrate levels of testosterone (<50 ng/dl) due to orchiectomy or luteinising hormone-releasing hormone (LHRH) agonist, undergoing ADT (gonadotropin-releasing hormone (GnRH) agonist/antagonist or prior bilateral orchiectomy)), and capable of performing exercise were recruited by clinician referrals.

Patients were randomly allocated to supervised exercise (EX) or a self-directed exercise control group (CON). The current study examines the initial 6 months of resistance and aerobic training completed thrice weekly as previously described [6], as the protocol for INTERVAL GAP4 initially commences with full supervision before transitioning to home-based exercise. Briefly, in the first and third exercise session of the week, structured resistance exercise (6 exercises, 2–5 sets, 6–12 RM intensity adjusted using repetition maximum (RM)) with a combination of high-intensity interval training (HIIT) (aerobic exercise, 6 x 60 s, intensity adjusted to a rating of perceived exertion (RPE) of 8 on 0–10 Borg scale) was prescribed, and 30–40 min of moderate-intensity continuous aerobic training (MICT) (cycling/walking) was undertaken at an intensity adjusted to RPE 6 in the second exercise session of the week. The exercise program was periodised and autoregulated across the week, month and 3-month cycles and autoregulated so that intensity, volume and exercise selection was adjusted depending on the patient’s readiness on the day. CON were provided with the American College of Sports Medicine (ACSM) guidelines for cancer survivors [36]. The study was funded by the Movember Foundation and ethically approved by the Human Research Ethics Committee at Edith Cowan University (ID: 13236 NEWTON). Written informed consent was obtained from all patients before inclusion.

### Body composition

Body composition was assessed at baseline and after the initial 6 months of the study by Dual-energy X-ray Absorptiometry (DXA; Horizon A, Hologic, Washington, USA). Values derived were whole-body lean mass (LM, kg), upper-body LM (kg), lower-body LM (kg), whole-body fat mass (FM, kg), percent LM, percent FM, and LM index (total lean mass/height squared; kg/m^2^). Body mass index (BMI) was calculated from weight divided by height squared (kg/m^2^).

### Blood assessment and analysis

Resting blood samples were collected early in the morning for fasting specimens and at least 48 h post any exercise. The collected blood samples were processed to serum and stored at −80 °C until serum myokine analysis. Serum myokine levels for irisin, IL-6, IL-15, SPARC, OSM, and myostatin, were analysed using multiplex magnetic bead panels (HMYOMAG-56k-15 Huma, Millipore, Billerica, MA, USA), and serum decorin (ab99998, Decorin Human ELISA Kit, Abcam, Cambridge, United Kingdom), IGF-1 (ab211651, Human IGF-1 SimpleStep ELISA Kit, Abcam, Cambridge, United Kingdom), and IGFBP-3 (ab211652, Human IGFBP3 SimpleStep ELISA Kit, Abcam, Cambridge, United Kingdom) levels were analysed using appropriate enzyme-linked immunosorbent assay (ELISA) kits.

### Cell culture and real-time cellular analysis

The human prostate cancer cell line, DU145 (ATCC HTB-81), was obtained from The Harry Perkins Institute for Medical Research, Nedlands, WA, Australia. Cells were cultured in RPMI-1640 media containing 10% fetal bovine serum (FBS), incubated at 37 °C, 5% CO2, and routinely passaged at ~80% confluence. Growth of DU145 cells was assessed using a Real-Time Cellular Analysis (RTCA) system, xCELLigence DP unit and E-plate (ACEA Bioscience, CA, USA) in the presence of human serum. Each well of E-plate was seeded with 15,000 DU145 cells with 100 µl of serum-free RPMI-1640. After 24 h of starvation, 100 µl of growth media (RPMI-1640) containing 20% human serum (final concentration of 10%) was added to each well of the E-plate. The plates were incubated for 72 h while recording the Cell Index every hour.

### Statistical analysis

Based on Cell Index results from our previous research (Pre: 5.829 ± 1.112; Post: 4.566 ± 1.515) [14], 24 participants (12 each group) is required to achieve 0.80 power at an α level 0.05 two-tailed. As a result, we obtained data and samples for 25 participants (CON, *n* = 12; EX, *n* = 13) from the INTERVAL GAP4 trial investigating the effect of exercise treatment on patients with mCRPC (Fig. 1). Data were analysed using R software (v4.0.2, The R Foundation), with the rstatix packages (v0.7.0, Kassambra, 2021) for statistics, ggplot2 packages (v3.3.3, Wickham, 2020) for visualisation and Desctools (v0.99.41, Slgnorell, 2021) for the area under the curve (AUC) calculations. Normality of the distribution for outcomes was tested using the Shapiro–Wilk test and Q-Q plot. Analysis of covariance (ANCOVA) was used to detect differences in post-intervention outcomes after 6 months for baseline value [37]. All values are presented as adjusted mean and 95% confidence interval. Tests were two-tailed and significance was set at *p* < 0.05.

## Results

### Patient characteristics

Patient characteristics are presented in Table 1. No significant difference between groups at baseline was evident in body weight, total LM, percent LM, LM index, FM, percent FM, BMI, serum levels of three myokines (OSM, SPARC, and decorin), and IGF-1, although serum levels of IGFBP-3 were significantly higher in CON. The average percentage of exercise sessions completed in EX was 82.5 ± 13.0% out of a total of 72 sessions.

**Table 1:** Baseline characteristics of exercise and usual care control group.

	CON (*n* = 12) (mean ± SD)	EX (*n* = 13)	*P*-value
		(mean ± SD)	
Age	76.9 ± 7.1	72.6 ± 7.0	0.140
Height (m)	1.7 ± 0.1	1.7 ± 0.1	0.608
Body weight (kg)	82.1 ± 13.4	93.7 ± 20.8	0.164
Total lean mass (kg)	49.1 ± 8.2	53.1 ± 10.4	0.364
Percent lean mass (%)	59.8 ± 4.0	57.0 ± 3.9	0.099
Lean mass index (kg/m^2^)	16.7 ± 2.1	17.6 ± 1.9	0.292
Total fat mass (kg)	26.9 ± 6.7	33.4 ± 10.5	0.118
Percent fat mass (%)	34.4 ± 4.7	37.1 ± 4.4	0.143
Presence of nodal metastasis	9	8	–
Presence of bone metastasis	8	10	–
Presence of nodal and bone metastasis	5	5	–
ARTA (e.g., abiraterone and enzalutamide) naïve patients	8	7	–
Patients on ARTA	4	6	–
BMI (kg/m^2^)	28.0 ± 4.0	31.1 ± 4.6	0.149
IGF-1 (ng/ml)	841.1 ± 636.6	806.5 ± 547.0	0.905
IGFBP-3 (ng/ml)	13273.6 ± 5871.5	6987.9 ± 2054.7	<0.001
Oncostatin M (ng/ml)	6.6 ± 4.9	4.5 ± 3.0	0.503*
SPARC (pg/ml)	495.7 ± 158.4	408.1 ± 82.1	0.605*
Decorin (ng/ml)	64.7 ± 7.3	63.0 ± 10.6	0.695

Three patients from the CON and EX group each commenced chemotherapy (Docetaxel or Cabazitaxel) during the exercise period. *ARTA* Androgen receptor-targeted agents. *Indicates Wilcoxon-rank test used for statistical analysis otherwise independent *t*-test.

### Body composition

Adjusted body weight, total LM, percent LM, LM index, FM, percent FM, and BMI adjusted for baseline did not show a significant difference between the EX and CON groups after the 6-month intervention (Table 2).

**Table 2:** Adjusted physical outcomes and serum OSM, SPARC, decorin, IGF-1, and IGFBP-3 levels. All outcomes are for the initial 6-month phase adjusted for baseline.

	CON (*n* = 12)		EX (*n* = 13)		*P*-value
	Adjusted mean	95% confidence interval	Adjusted mean	95% confidence interval	
Physical Outcomes					
Body weight (kg)	89.5	[87.6, 91.5]	86.7	[84.9, 88.6]	0.052
Total lean mass (kg)	50.7	[49.4, 51.9]	50.6	[49.4, 51.8]	0.948
Percent lean mass (%)	57.7	[56.4, 59.0]	58.4	[57.1, 59.6]	0.454
Lean mass index (kg/m^2^)	17.0	[16.6, 17.4]	17.2	[16.8, 17.6]	0.625
Total Fat Mass (kg)	32.1	[30.0, 34.1]	29.8	[27.9, 31.8]	0.126
Percent Fat Mass (%)	36.7	[35.1, 38.2]	35.9	[34.4, 37.5]	0.513
BMI (kg/m^2^)	30.0	[29.4, 30.7]	29.2	[28.6, 29.8]	0.068
Serum IGF-1, IGFBP-3, and myokine levels					
* IGF-1 (ng/ml)*	924.85	[687.47, 1162.24]	878.29	[650.23, 1106.36]	0.772
* IGFBP-3 (ng/ml)*	10186.76	[8554.44, 11819.08]	7884.04	[6329.68, 9438.40]	0.068
* IGF-1:IGFBP-3 ratio*	0.10	[0.05, 0.15]	0.14	[0.09, 0.19]	0.307
* OSM (ng/ml)*	4.88	[2.17, 7.59]	8.71	[6.11, 11.30]	0.050
* SPARC (pg/ml)*	410.58	[362.18, 458.97]	492.66	[446.28, 539.04]	0.022
* Decorin (ng/ml)*	67.08	[62.92, 71.23]	63.75	[59.76, 67.74]	0.246
* Relative IGF-1 (ng/m/kg)*	11.25	[8.31, 14.19]	10.17	[7.34, 12.98]	0.586
* Relative IGFBP-3 (ng/ml/kg)*	120.40	[101.35, 139.45]	97.71	[79.57, 115.84]	0.120
* Relative OSM (ng/ml/kg)*	0.06	[0.03, 0.09]	0.10	[0.07, 0.13]	0.083
* Relative SPARC (pg/ml/kg)*	4.85	[4.32, 5.37]	5.73	[5.22, 6.23]	0.025
* Relative Decorin (ng/ml/kg)*	0.78	[0.74, 0.83]	0.77	[0.73, 0.82]	0.770

All 6-month outcomes are adjusted for the baseline value.

### Myokines and IGF-1/IGFBP-3

A total of seven different myokines (irisin, decorin, IL-6, IL-15, SPARC, OSM, and myostatin), IGF-1, and IGFBP-3 were analysed from serum acquired at baseline and after the exercise intervention to examine the effect of exercise on serum levels of myokines and the IGF-1 axis. However, due to a low recovery rate of irisin, IL-6, IL-15, and myostatin in multiplexed magnetic bead-based immunoassay, only OSM, SPARC, decorin, IGF-1, and IGFBP-3 were able to be analysed (Table 2). After adjusting 6-month-intervention serum levels for baseline levels, no significant differences were observed in IGF-1, IGFBP-3 levels, or IGF-1/IGFBP-3 ratio. However, there were significant differences between groups in serum levels of OSM (*P* = 0.050) and SPARC (*P* = 0.022) at post-intervention adjusted by baseline. Relative SPARC levels (serum SPARC levels/body weight) increased significantly in EX compared to CON (*P* = 0.025), and there was a trend for an increase in relative OSM (serum OSM levels/body weight) (*P* = 0.083).

Subgroup analysis for ARTA (CON-ARTA naïve, *N* = 8; CON-ARTA, *N* = 4; EX-ARTA naïve, *N* = 7; EX-ARTA, *N* = 6) demonstrated a significant increase in adjusted serum OSM and SPARC levels for baseline in EX-ARTA naïve compared to the patients in CON-ARTA (*P* < 0.05; Supplementary Table 1S). In addition, subgroup analysis by chemotherapy (CON-NO Chemo, *N* = 9, CON-Chemo, *N* = 3; EX-NO Chemo, *N* = 10, EX-Chemo, *N* = 3), revealed baseline adjusted serum OSM and SPARC levels to be significantly higher in EX Chemo group compared to CON Chemo group (*P* < 0.05; Supplementary Table 2S). Furthermore, baseline adjusted serum decorin levels were significantly higher in the EX-ARTA subgroup compared to EX-ARTA naïve subgroup (*P* < 0.05; Supplementary Table 1S), however, significantly lower baseline adjusted decorin was observed in the EX-Chemo subgroup compared to CON-NO Chemo subgroup (*P* < 0.05; Supplementary Table 2S).

### Prostate cancer cell line growth analysis

**Fig. 2:**
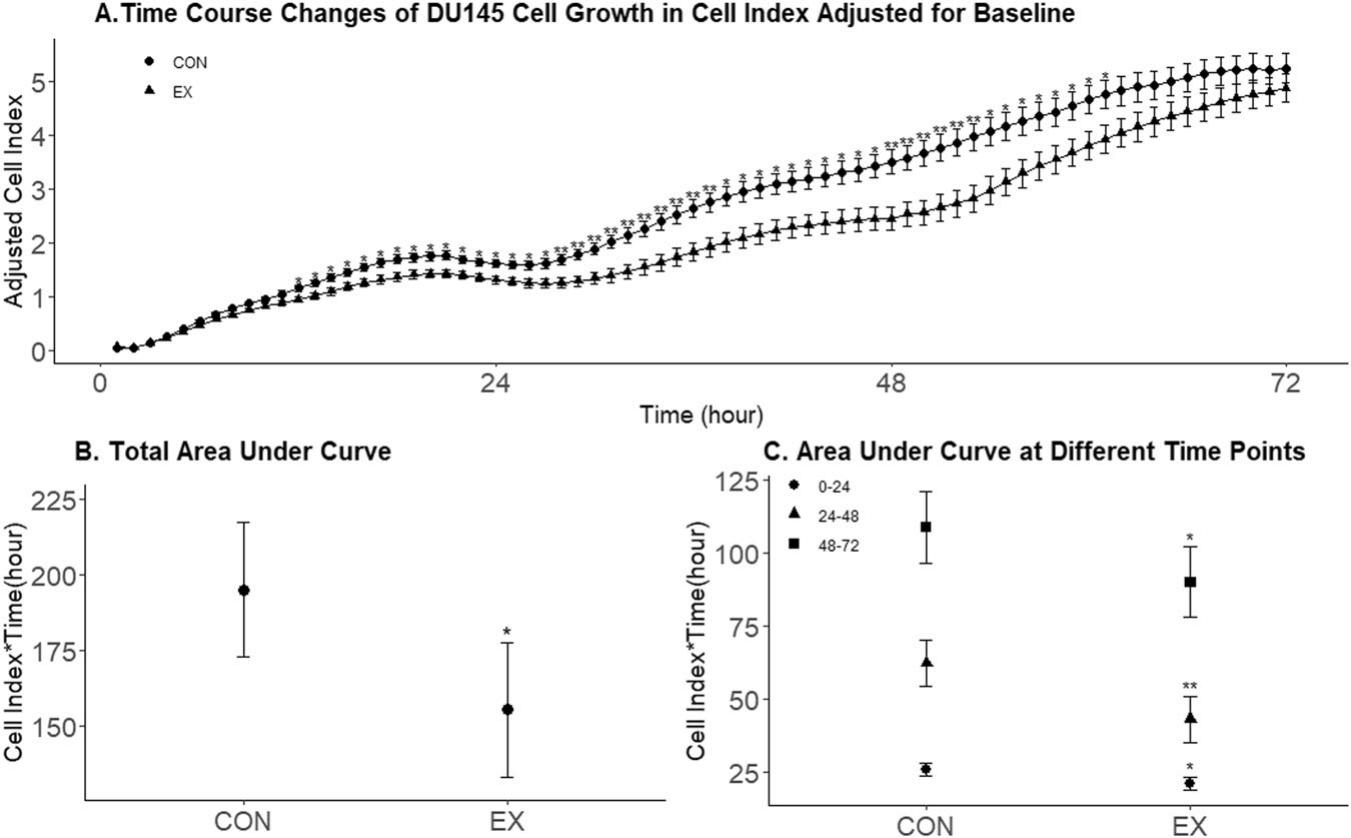
Cell proliferation data. **A** Time-course changes of DU145 Cell growth in Cell Index adjusted for baseline values at each time point (adjusted mean±adjusted SE). The error bar indicates an adjusted SE. **B** Adjusted Area Under Curve (AUC) over 72 h incubation. **C** Adjusted Area Under Curve at different time points (0 to <24 h, 24 to < 48 h, 48 to 72 h). Due to contamination of a serum sample from one patient in the EX group, serum samples from only 12 patients in the CON group and 12 patients in the EX group were analysed. The grey bar indicates a 95% confidence interval of adjusted values. *P < 0.05, **P < 0.01.

Three-day cell growth kinetics are presented in Fig. 2A. Although adjusted Cell Index at 72 h after administrating 6-month serum from EX or CON groups did not show a difference, adjusted Cell Index at 12 to 61 h revealed a significant decrease in EX compared to CON at each time point. Therefore, the area under curve (AUC) was calculated from the 72 h Cell Index plot (Table 3, Fig. 2B, C). Total cell growth AUC (0–72 h) was significantly reduced with the presence of serum obtained after 6 months of an exercise intervention in EX compared to CON after adjusting for baseline AUC (*P* = 0.029). In addition, the adjusted 6-month timepoint intervention AUC at 0 to < 24 h, 24 to < 48 h and 48 to 72 h periods exhibited a significant decrease in EX compared to CON (*P* = 0.016, *P* = 0.006, and *P* = 0.039, respectively) (Table 3, Fig. 2C).

**Table 3:** Area under curve from adjusted Cell Index adjusted for baseline.

Time frame (hour)	CON (*n* = 12)		EX (*n* = 12)		*P*-value
	Adjusted mean (Cell Index*Time(h))	95% confidence interval	Adjusted mean (Cell Index*Time(h))	95% confidence interval	
0–72	195.00	[172.71, 217.28]	155.34	[133.05, 177.62]	0.029
0–24	25.72	[23.40, 28.03]	20.87	[18.55, 23.18]	0.016
24–48	62.06	[54.19, 69.94]	43.05	[35.17, 50.92]	0.006
48–72	108.83	[96.65, 121.01]	90.06	[77.85, 102.24]	0.039

The 6-month AUC outcomes are adjusted for baseline AUC. Due to contamination of a serum sample from one patient in the EX group, serum samples from only 12 patients in the CON group and 12 patients in the EX group were analysed.

## Discussion

In this sub-study comprised from the INTERVAL-GAP4 randomised controlled trial [6], we examined chronic adaptations in potential muscle-induced candidates for tumour suppression in men with mCRPC undertaking 6 months of exercise training. We confirmed that the first 6 months of an exercise intervention vs control produced an increase in myokine expression, specifically OSM, SPARC, and body weight relative SPARC, as well as a borderline increase in relative OSM. We also examined the effect of serum collected at baseline and 6 months on prostate cancer cell line DU145 growth. Although we cannot definitely conclude a direct relationship between exercise training elevated myokines and prostate cancer cell line growth, the 6-month serum drawn from the EX reduced DU145 cancer cell growth compared to CON.

While there are numerous hypotheses, the mechanistic details by which exercise influences tumour biology are unknown. In our prior review [5], we provide a rationale for the influence of muscle-induced myokines as anti-cancer agents acting through several pathways to drive apoptosis and suppress proliferation and metastasis. Furthermore, our team recently completed a single-group trial involving prostate cancer patients receiving ADT, and we demonstrated positive alterations in myokine concentrations with subsequent growth suppression of prostate cancer cell lines [14]. While this finding is intriguing, the question remained whether such anti-cancer effects of exercise could be induced in patients with more advanced prostate cancer. Patients with mCRPC have a very high disease load combined with accumulated treatment toxicities, resulting in considerable deconditioning, reduced muscle and bone mass and elevated fat mass. Should an anti-cancer systemic mechanism produced through exercise therapy be evident, then this could be a particularly attractive strategy for patients with mCRPC to slow disease progression.

The current study is the first to examine myokine expression before and at the 6-month exercise intervention in patients with mCRPC incorporating resistance and aerobic training. We focused on the endocrine function of skeletal muscle [15], given that preclinical studies demonstrated a positive role of myokines in cancer cell suppression [19, 20, 24–27, 30] and a retrospective study in prostate cancer patients showed a positive association between skeletal muscle mass and progression-free survival [38]. The results of serum myokine analysis in the current study showed significant elevation of serum SPARC and OSM levels in the EX group compared to the CON group, which is consistent with our previous report [14] in predominantly localised prostate cancer patients.

In addition, although this is speculative due to the limited sample number, our subgroup analysis for different treatments provided interesting observations regarding myokine expression. As androgen is a critical factor in muscle growth, we expected ARTA (androgen receptor-targeted agent), commonly prescribed as the first-line treatment for castrate-resistant prostate cancer, may have an impact on exercise-induced serum myokine levels. Interestingly, our subgroup analysis for ARTA and ARTA naïve showed a significant increase of serum OSM and SPARC in the EX-ARTA naïve subgroup compared to the CON-ARTA subgroup, suggesting that ARTA may impact exercise-induced OSM and SPARC levels possibly due to ARTA impacts on muscle size and physiology. Furthermore, our ARTA subgroup analysis demonstrated significantly increased serum decorin levels in the EX-ARTA group compared to EX-ARTA naïve; however, a decreased trend of decorin levels was observed in EX-Chemo compared to EX-NO Chemo, suggesting exercise-induced alteration of serum decorin may partially be impacted by chemotherapy. However, although these observations from our subgroup analysis are noteworthy, further research with larger patient numbers is required to fully elucidate these interactions.

Given that body composition, in terms of fat and muscle mass, influences cytokine levels in the blood [39, 40], it was somewhat surprising that we did not observe improvements in body composition. Although the intensity and volume prescribed to these patients were considerably high, a lack of differential response may be due to the disease and treatment load these patients are experiencing, compromising their ability to adapt with morphological changes. Similarly, a lack of change in body composition was also evident in our previous study involving prostate cancer patients with bone metastases following a 3-month exercise intervention [41]. The interference of aerobic exercise on adaptations to resistance training may also have occurred in these patients with mCRPC, as we have reported previously for men with nonmetastatic prostate cancer being treated with ADT [42]. Whether or not this impacted the magnitude of increase in myokines and subsequent growth suppression in the cell line experiments cannot be determined.

We also observed a reduced prostate cancer cell line (DU145) growth after directly applying resting serum acquired following 6 months of exercise. For clinical relevance, we recruited patients under very strict criteria [6] and used the androgen-insensitive, metastatic prostate cancer cell line DU145 as this cell line originated from a 69-year-old Caucasian male with metastatic prostate cancer, which shares similar characteristics with mCRPC. Furthermore, it should be noted that resting serum was collected after at least 48 h of complete rest, and the acute physiological arousal from the last exercise session did not affect the results. Our previous report also observed reduced prostate cancer cell line growth by directly applying resting exercise-conditioned serum acquired from predominantly localised prostate cancer patients [14], suggesting exercise adaptation-induced systemic milieu alteration might positively influence tumour biology. Furthermore, previous studies by Barnard et al. [7], Leung et al. [11], and Ngo et al. [12] also reported reduced prostate cancer cell line (LNCaP) growth with the presence of resting serum obtained from active, healthy individuals and healthy persons following a short-term period of exercise and dietary intervention. Although these reports demonstrated the potential role of exercise in inducing changes in the IGF-1 axis [7, 11, 12], serum IGF-1 and IGFBP-3 levels did not change in our cohort, suggesting that the serum myokine level changes due to exercise training are more likely candidate drivers suppressing DU-145 growth rather than the IGF-1 axis. Nevertheless, consistent reduction of prostate cancer cell growth in previous studies and the current study provides important insight in the field of exercise oncology that not only should exercise be considered as a strategy to improve health-related outcomes for prostate cancer patients, but also as a potential daily-dosage strategy to create a tumour-suppressive environment.

The current study has a number of strengths and limitations worthy of comment. First, we used a randomised control trial design to investigate the expression of multiple myokines resulting from exercise. Second, DXA was used for body composition assessment providing accurate measures of fat and lean tissue. Third, by using RTCA, we were able to detect cell growth differences in multiple time points. This is important as previous studies that observed cell growth after applying human serum used end-point analysis and were unable to monitor cell growth kinetics. Fourth, the study provided clinically relevant evidence for tumour suppression using serum acquired from prostate cancer patients with mCPRC as prior to this data for myokine expression research has been limited to either healthy cohorts or those with metabolic disease, or patients with less advanced disease. However, as we used a multimodal exercise program, we cannot determine which exercise mode or if both contributed to the adaptation in myokine expression. Moreover, we analysed the data and samples available in our ongoing trial, INTERVAL-GAP4, which limits the volume of serum available for individual experiments and so this study was confined to investigate serum levels of myokines and prostate cancer cell growth. As there is limited research investigating myokine expression in patients with advanced prostate cancer, we made our initial sample size and power calculation based on available data from our previous trial in prostate cancer patients with localised disease [14]. Unfortunately, for the current study in patients with advanced disease our post-hoc analysis revealed that we only achieved statistical power of 65% in the adjusted AUC of Cell index, which likely reflects differences between the two patient groups in exercise responses. In addition, although we reported an increase in serum myokine levels and a significant reduction of DU145 cell growth after applying exercise-conditioned human serum, the current study is limited with regard to in-depth intercellular mechanistic measures to address the tumour-suppressive role of exercise-induced myokines or potential interaction between the treatments and myokines.

In conclusion, this study provides preliminary evidence for enhanced myokine expression, and a tumour-suppressive effect of serum collected from mCRPC patients after 6 months of vigorous, multimodal exercise. Future trials are needed to further elucidate the influence of exercise on myokine expression, particularly specifics of exercise prescription such as threshold exercise intensity, volume, and mode. Furthermore, more in-depth intercellular mechanistic research involving the application of both acute and chronically exercise-conditioned human serum is required to enhance our understanding of the direct tumour-suppressive role of myokines in patients with prostate cancer.

## Supplementary information

Supplementary tables

## Data Availability

The data are available for bona fide researchers who request it from the authors.
