# Supplementary material for: Exercise in advanced prostate cancer elevates myokine levels and suppresses in-vitro cell growth
Source: Prostate Cancer Prostatic Dis. 2022 Feb 12;25(1):86–92. doi: 10.1038/s41391-022-00504-x (PMC8853098; doi:10.1038/s41391-022-00504-x)
Supplement: Supplementary file 1 — Supplementary tables [file 41391_2022_504_MOESM1_ESM.docx]

Supplementary Table 1S. Subgroup analysis for serum myokine levels adjusted for baseline (ARTA).

|  | CON (N=12) | | | | EX (N=13) | | | | p-value |
| --- | --- | --- | --- | --- | --- | --- | --- | --- | --- |
|  | ARTA naïve (n=8) | | ARTA (n=4) | | ARTA naïve (n=7) | | ARTA (n=6) | |  |
|  | Adjusted mean | 95% confidence interval | Adjusted mean | 95% confidence interval | Adjusted mean | 95% confidence interval | Adjusted mean | 95% confidence interval |  |
| *IGF-1 (ng/ml)* | 1009.58 | [716.78, 1302.38] | 756.10 | [330.03, 1182.16] | 1004.88 | [688.18, 1321.58] | 730.15 | [383.27, 1077.31] | 0.123 |
| *IGFBP-3 (ng/ml)* | 11251.27 | [9378.16, 13124.44] | 8410.92 | [6020.51, 10801.34] | 8306.74 | [6432.17, 10181.31] | 7155.40 | [5094.78, 9216.03] | 0.315 |
| *IGF-1:IGFBP-3 ratio* | 0.09 | [0.02, 0.16] | 0.09 | [0.01, 0.18] | 0.17 | [0.09, 0.25] | 0.11 | [0.04, 0.18] | 0.115 |
| *OSM (ng/ml)* | 5.41 | [2.08, 8.75] | 3.78 | [0, 9.03] | 9.87† | [6.32, 13.42] | 7.37 | [3.35, 11.39] | 0.216 |
| *SPARC (pg/ml)* | 425.88 | [368.87, 482.88] | 380.23 | [300.48, 459.99] | 523.60† | [463.07, 584.14] | 456.37 | [390.98, 521.78] | 0.04 |
| *Decorin (ng/ml)* | 67.69 | [63.34, 72.05] | 66.01 | [59.88, 72.15] | 58.81 | [54.12, 63.51] | 69.40* | [64.38, 74.42] | 0.02 |
| *Relative IGF-1(ng/m/kg)* | 12.23 | [8.60, 15.88] | 9.10 | [3.36, 14.84] | 11.30 | [7.46, 15.15] | 8.94 | [4.61, 13.27] | 0.565 |
| *Relative IGFBP-3(ng/ml/kg)* | 129.38 | [108.37, 150.39] | 100.28 | [70.59, 129.97] | 105.77 | [83.56, 128.19] | 89.74 | [65.79, 113.69] | 0.267 |
| *Relative OSM*  *(ng/ml/kg)* | 0.07 | [0.03, 0.11] | 0.04 | [0, 0.10] | 0.02 | [0.07, 0.15] | 0.09 | [0.04, 0.14] | 0.182 |
| *Relative SPARC (pg/ml/kg)* | 5.03 | [4.44, 5.63] | 4.35 | [3.46, 5.24] | 6.10† | [5.44, 6.76] | 5.37 | [4.69, 6.05] | 0.03 |
| *Relative Decorin (ng/ml/kg)* | 0.79 | [0.74, 0.84] | 0.77 | [0.70, 0.85] | 0.73 | [0.67, 0.78] | 0.83 | [0.77, 0.88] | 0.123 |

*indicates EX-ARTA naïve vs. EX-ARTA at baseline with in the group; †indicates CON-ARTA at baseline vs. EX-ARTA naïve.

Supplementary Table 2S. Subgroup analysis for serum myokine levels adjusted for baseline (Chemo).

|  | CON (N=12) | | | | EX (N=13) | | | | p-value |
| --- | --- | --- | --- | --- | --- | --- | --- | --- | --- |
|  | NO Chemo (n=9) | | Chemo (n=3) | | NO Chemo (n=10) | | Chemo (n=3) | |  |
|  | Adjusted mean | 95% confidence interval | Adjusted mean | 95% confidence interval | Adjusted mean | 95% confidence interval | Adjusted mean | 95% confidence interval |  |
| *IGF-1 (ng/ml)* | 952.48 | [659.36, 1245.59] | 840.34 | [307.78, 1372.90] | 879.64 | [606.04, 1153.24] | 239.38 | [376.11, 1374.79] | 0.974 |
| *IGFBP-3 (ng/ml)* | 10436.66 | [8593.42, 12279.88] | 9195.306 | [6922.93, 12467.68] | 7986.33 | [6161.07, 9811.58] | 7784.86 | [4742.02, 10827.69] | 0.293 |
| *IGF-1:IGFBP-3 ratio* | 0.10 | [0.04, 0.16] | 0.10 | [0, 0.20] | 0.13 | [0.07, 0.19] | 0.15 | [0.05, 0.26] | 0.762 |
| *OSM (ng/ml)* | 6.02 | [3.31, 8.73] | 1.93 | [0, 6.56] | 7.04 | [4.43, 9.66] | 13.79** | [9.16, 18.42] | 0.01 |
| *SPARC (pg/ml)* | 423.90 | [368.97, 478.82] | 377.90 | [288.63, 467.16] | 498.41 | [418.47, 518.36] | 566.19** | [477.15, 655.23] | 0.02 |
| *Decorin (ng/ml)* | 67.69 | [63.39, 71.99] | 65.44 | [58.03, 72.85] | 66.33 | [62.29, 70.37] | 54.95* | [47.50, 62.40] | 0.04 |
| *Relative IGF-1 (ng/m/kg)* | 11.41 | [7.84, 14.98] | 10.68 | [3.53, 17.83] | 10.19 | [6.78, 13.59] | 10.16 | [4.03, 19.28] | 0.957 |
| *Relative IGFBP-3 (ng/ml/kg)* | 122.03 | [100.91, 143.16] | 111.75 | [70.41, 153.09] | 100.05 | [78.57, 121.53] | 17.23 | [57.71, 129.58] | 0.447 |
| *Relative OSM (ng/ml/kg)* | 0.07 | [0.04, 0.11] | 0.015 | [0, 0.07] | 0.08 | [0.05, 0.12] | 0.15** | [0.10, 0.21] | 0.017 |
| *Relative SPARC (pg/ml/kg)* | 5.03 | [4.45, 5.61] | 4.31 | [3.36, 5.27] | 5.53 | [4.99, 6.07] | 6.37** | [5.40, 7.34] | 0.031 |
| *Relative Decorin (ng/ml/kg)* | 0.78 | [0.74, 0.83] | 0.79 | [0.71, 0.87] | 0.80 | [0.76, 0.85] | 0.68† | [0.59, 0.76] | 0.070 |

*indicates CON-NO Chemo vs. EX Chemo; **indicates CON Chemo vs. EX Chemo; †indicates EX Chemo vs. EX NO Chemo, p=0.060.
